# Hepatitis a virus infection in Central-West Tunisia: an age structured model of transmission and vaccination impact

**DOI:** 10.1186/s12879-020-05318-7

**Published:** 2020-08-26

**Authors:** Kaouther Ayouni, Bechir Naffeti, Walid Ben Aribi, Jihène Bettaieb, Walid Hammami, Afif Ben Salah, Hamadi Ammar, Slimane Ben Miled, Henda Triki

**Affiliations:** 1 Laboratory of Clinical Virology – Pasteur Institute of Tunis, University of Tunis El Manar, 13, Place Pasteur, BP: 74-1002, Tunis, Tunisia; 2 Clinical Investigation Center - Pasteur Institute of Tunis, University of Tunis El Manar, Street 15 Medenine Bardo, Tunis, Tunisia; 3 Faculty of Sciences of Tunis, University of Tunis El Manar, Campus Universitaire 2092-El Manar, Tunis, Tunisia; 4 Laboratory of Intelligent Networks and Nanotechnology, LARINA, University of Carthage, Tunis, Tunisia; 5 Faculty of Sciences of Bizerte, University of Carthage, Avenue de la République, P. O. Box 77-1054, Tunis, Amilcar Tunisia; 6 Department of Epidemiology - Clinical Investigation Center - Pasteur Institute of Tunis, University of Tunis El Manar, 13, Place Pasteur, BP: 74-1002, Tunis, Tunisia; 7 Faculty of Medicine of Tunis, University of Tunis El Manar, 15 Rue Djebel Lakhdhar. La Rabta, 1007 Tunis, Tunisia; 8 Faculty of Economic Sciences and Management of Nabeul, University of Carthage, Avenue de la République, BP 77-1054, Tunis, Amilcar Tunisia; 9 Laboratory of Bioinformatics, Biomathématics and Biostatistics, Pasteur Institute of Tunis, University of Tunis El Manar, 13, Place Pasteur, BP: 74-1002, Tunis, Tunisia

**Keywords:** Hepatitis A virus, Seroprevalence, Vaccination, Public health, Mathematical model

## Abstract

**Background:**

The epidemiological pattern of hepatitis A infection has shown dynamic changes in many parts of the world due to improved socio-economic conditions and the accumulation of seronegative subjects, which leads to possible outbreaks and increased morbidity rate. In Tunisia, the epidemiological status of hepatits A virus is currently unknown. However, over the past years higher numbers of symptomatic hepatitis A virus infection in school attendants and several outbreaks were reported to the Ministry of Health, especially from regions with the lowest socio-economic levels in the country. The aim of this study was to investigate the current seroprevalence of hepatitis A virus antibodies in central-west Tunisia and assess the impact of hepatitis A virus vaccination on hepatitis A epidemiology.

**Methods:**

Serum samples from 1379 individuals, aged 5–75 years, were screened for hepatitis A virus antibodies. Adjusted seroprevalence, incidence and force of infection parameters were estimated by a linear age structured SEIR (Susceptible-Exposed-Infectious-Recovered) compartmental model. A vaccine model was then constructed to assess the impact on hepatitis A virus epidemiology of 3 scenarios of vaccination strategies: one dose at 12-months of age, one dose at 6-years and one dose at 12-months and another at 6-years of age during 6 years.

**Results:**

A rapid increase in anti-hepatitis A virus seroprevalence was noted during infancy and adolescence: 47% of subjects under 10-years-old are infected; the prevalence increases to 77% at 15-years and reaches 97% in subjects aged 30-years. The force of infection is highest between 10 and 30-years of age and the incidence declines with increasing age. The vaccine model showed that the 3-scenarios lead to a significant reduction of the fraction of susceptibles. The two doses scenario gives the best results. Single-dose vaccination at 6-years of age provides more rapid decrease of disease burden in school-aged children, as compared to single-dose vaccination at 12-months, but keeps with a non-negligible fraction of susceptibles among children < 6-years.

**Conclusions:**

Our study confirms the epidemiological switch from high to intermediate endemicity of hepatitis A virus in Tunisia and provides models that may help undertake best decisions in terms of vaccinations strategies.

## Background

Hepatitis A virus (HAV) is a non-enveloped RNA Picornavirus, responsible annually for almost 1.5 million cases of acute hepatitis [1]. It is the most common cause of acute viral hepatitis worldwide, causing substantial morbidity, with tens of millions of infected cases worldwide [2]. However, the true incidence of the disease remains underestimated due to the high frequency of asymptomatic forms and of symptomatic forms that are not reported [3]. Hepatitis A virus is mainly transmitted through fecal-oral route either by direct contact of a susceptible person with an infectious person (generally through contaminated hands) or by ingestion of contaminated food or water [2]. It usually causes a self-limiting liver infection, especially in children, with a benign clinical course and no evolution to chronicity. It may occasionally progress to severe disease, especially among elderly population [3, 4].

Different geographical patterns of HAV infection exist, correlating with sanitary conditions and development indicators [5]. The infection occurs more frequently in populations of less economically-developed regions with little education and poor hygiene [6]. Due to improved socio-economic conditions, the epidemiological pattern of hepatitis A infection recently changed in many parts of the world, including Asia, Latin America, Eastern Europe and the Middle East [2, 7–16]. Although this represents a positive indicator for socio-economic development, it leads to possible outbreaks due to the accumulation of seronegative subjects [17, 18] and increased morbidity rate as a result of the increased age of primary infection.

According to published data from the ‘90s; Tunisia counts among high endemic countries and most cases occur in young children [19]. Due to the progress in sanitation and socio-economic conditions, the epidemiology of hepatitis A has shown dynamic changes over the past years. A decline in the seroprevalence of HAV in children and teenagers was reported in more recent studies which suggests that the epidemiology of HAV in Tunisia is changing from high to intermediate endemicity [20, 21]. Rezig et al. [21] also showed lower seroprevalences in the major cities of the northern and eastern coasts as compared to regions located in the southern and central- western parts of the country which have lower socio-economic levels. In 2012, Khelifi et al., showed that the rate of acute infection increased significantly as compared to the rate reported at the beginning of the 1980s to reach 42% in patients over 15 years [22]. In 2014, Hellara et al., showed a higher rate of acute infection in patients aged 15–25 years [23]. From 2015 onwards, higher numbers of symptomatic HAV infection in school attendants and several outbreaks were reported to the Ministry of Health (unpublished data), especially from the regions with lowest socio-economic levels in the country. This represents a serious public health problem and several actions are now being considered by the sanitarian authorities to interrupt these outbreaks in school attendants among which the introduction of HAV vaccination in the National Program of Immunization. Taking into consideration budget limitations, the introduction of more than one dose of vaccine, in newborns and other ages, may not be possible and only one dose would be used. Hence, research studies assessing the possible impact of different vaccination strategies on the epidemiology of the disease can help the NITAG (National Immunization Technical Advisory Group) to choose the best vaccination strategies. Dynamic mathematical models are nowadays of widespread use and offer the possibility to assess infectious disease epidemiology dynamics after vaccine introduction. Mathematical models provide insight into HAV vaccine impact under various vaccination scenarios as well as its cost-effectiveness [24–27].

The aim of the present study was to investigate the current seroprevalence of HAV antibodies (anti-HAV) among residents in a rural setting in central-west Tunisia and to take this population as a model to assess the impact of the introduction of HAV vaccination on the epidemiology of the disease, using different vaccination scenarios. Therefore, we determined the age specific seroprevalences for HAV infection according to laboratory and demographic data. A first mathematical model, called “adjusted model”, was then constructed to estimate the adjusted seroprevalences, incidence and force of infection parameters. A second model, called “vaccine model”, was then constructed to predict the evolution of HAV infection according to various vaccination options. This second model extends the first model by integrating demographic parameters and contact rate between individuals. It uses the force of infection and the steady state of the first model as input parameters.

## Methods

### Study area, samples and data collection

The study was conducted in Thala, a predominantly rural area in the Central-West of Tunisia (governorate of Kasserine); covering 752.2 km^2^ with a population of 34,508 inhabitants; according to the 2014 national census. According to the Tunisian sanitary map (a report from the Ministry of Health on the sanitary infrastructure, human resources and health status by regions of the country), this region is classified among the areas of higher poverty in Tunisia together with other regions covering almost 23% of the country [28]. The study population comprised 1379 individuals, representing 5% of the target population and selected through a household cross-sectional survey conducted between January and June 2014. A multistage stratified random cluster sampling method was used to include all dwellings and their individuals from the 13 districts of Thala. Blood samples from the enrolled subjects aged 5–75 years were collected using sterile vacuum blood collection tubes. The samples collected were stored at + 4 °C and then transferred refrigerated and within 24 h to the laboratory of Clinical Virology at the Pasteur Institute of Tunisia where they were immediately centrifuged and the sera collected and stored at − 20 °C until serological testing.

### Laboratory testing

Sera obtained from the 1379 enrolled individuals were screened for IgG antibodies to HAV using a commercially available immunoassay kits: Monalisa™ Total Anti-HAV Plus (BIORAD, France), an indirect ELISA with a 100% specificity and 100% sensitivity as indicated by the manufacturer.

### Adjusted model for seroprevalence, incidence and force of infection

**Fig. 1:**
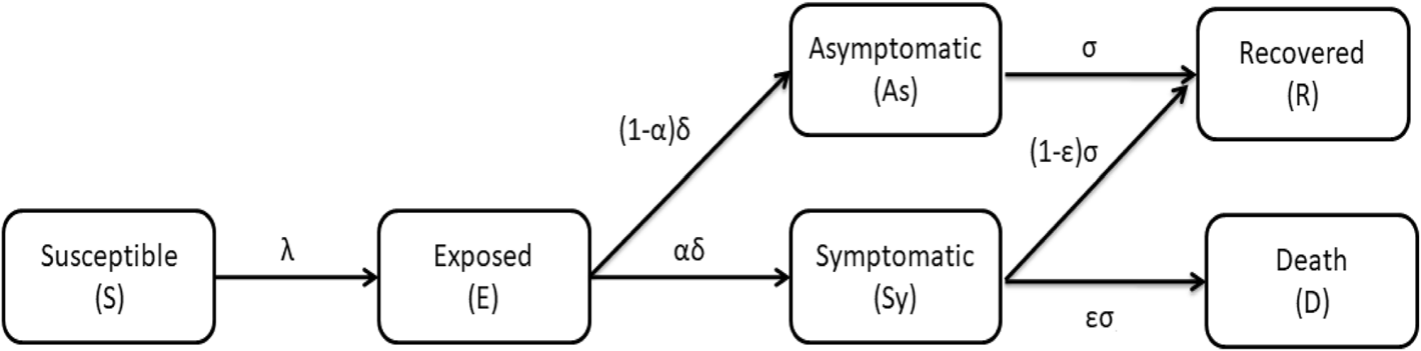
Conceptual diagram of the SEIR model. All individuals are in susceptible class (S). When acquiring infection individuals first transfer to the latent or exposed class (E) before becoming infectious, eventually individuals move to the immune class (R) and death class (D)

A linear age structured SEIR (Susceptible-Exposed-Infectious-Recovered) compartmental model (Fig. 1) was developed, where the infectious compartment is divided into symptomatic (Sy) and asymptomatic (As) compartments [24]. It was assumed that all individuals that are in the susceptible class remain there until they are exposed. HAV disease has a latent period of 1/ *δ*, exposed individuals (E) become infectious at rate *δ* and may develop symptoms or not. The severity of the disease is age-dependent; children under 6 years are usually asymptomatic, older children and adult usually have more severe symptoms i.e. older individuals are more likely to develop jaundice and fulminant hepatic failure. The rate of mortality by HAV disease increases from 0.1% for individuals < 15 years-old to 2.1% for individuals over 40 years-old. In this model, we consider that the force of infection (λ) is age-dependent, i.e. at rate λ, susceptible persons (S) may be exposed to the infection after a contact with the virus. Asymptomatic persons (As) will clear their infection and move to the recovered compartment (R). Symptomatic persons (Sy) may clear their infection and develop a lifelong immunity (i.e. move to the recovered compartment (R)) or die (D). The infection rate was estimated using seroprevalence data.

To simplify the model, we assumed that newborns enter the susceptible compartment at the age of 1 year (when the immunity derived from maternal antibodies wanes) and assume that the population number is constant through time. Let x(a), y(a), z(a), u(a), r(a) and w(a) are the fractions of susceptible, exposed, asymptomatic, symptomatic, recovered and dead persons.

The model is given by the following equations:

1 $$ {\displaystyle \begin{array}{c}\frac{dx}{da}=-\lambda (a)x(a)\\ {}\frac{dy}{da}=\lambda (a)x(a)-\delta y(a)\\ {}\frac{dz}{da}=\left(1-\alpha \right)\delta y(a)-\sigma (a)z(a)\\ {}\frac{du}{da}=\alpha \delta y(a)-\sigma (a)u(a)\\ {}\frac{dr}{da}=\sigma (a)z(a)+\left(1-\varepsilon (a)\right)\sigma (a)u(a)\\ {}\frac{dw}{da}=\varepsilon (a)\sigma (a)u(a)\end{array}} $$

Where at age 1,

$$ x(1)=1 $$

$$ y(1)=z(1)=u(1)=r(1)=w(1)=0 $$

and *λ* is the force of infection, *δ* is the mean rate at which an exposed individual becomes infectious and is the inverse of the mean latent period (1/*δ*), 1/*σ* is the average duration of infectivity by age, *ε* is the death rate due to HAV virus, *α* is the probability an infection is icteric.

The force of infection was estimated by the probability $\pi (a)=1-\mathit{\exp}\left(-{\int}_0^a\lambda (a) da\right)$, that is defined by the probability of being infected before age a which is parameterized using the data of anti-HAV seroprevalence in the study area [See Additional file 1].

### The epidemiologic model with vaccination (or vaccine model)

A second model, called vaccine model, was developed to assess the impact of several vaccination strategies on HAV evolution. This model, reformulates the adjusted model by integrating the time, t, and age of the individuals, noted i, to generate the fraction of susceptible, exposed, asymptomatic, symptomatic and immunes, under the assumption of time-dependent endemic equilibrium and with implementation of vaccine strategies [25].

**Fig. 2:**
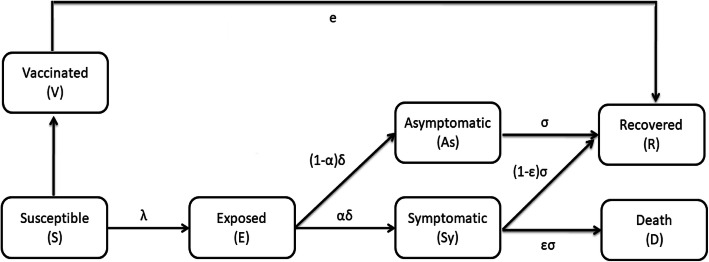
Conceptual diagram of Hepatitis A transmission and vaccination model. After infection, individual moves from the susceptible compartment (S) to the exposed compartment (E) and becomes infectious after a latent period. Asymptomatic individuals (As) can clear their infection and move to the recovered compartment (R). Symptomatic individuals (Sy) may clear their infection and develop a lifelong immunity (i.e. move to the recovered compartment (R)) or die (D). Upon vaccination, a fraction of susceptible individual (e) of ages a moves to the vaccinated compartment and becomes immune

This model was divided into two processes: the demographic processes (mortality and birth) and epidemiological processes. It is assumed without losing generality that the population is assumed to be constant in number and age distribution throughout the year. Demography is taken into account at the end of the year, where population density and age distribution are adjusted according to demography. Therefore, demography and epidemiological processes act at different time scale. The epidemiological time is denoted by t (in days) and demographic time by *τ* (in years). We suppose that the disease acts at a faster time scale than demography, *t* ≪ *τ* and that the demographic processes (natural birth and death) acts at the beginning of the year and then during the rest of the year the epidemiological dynamics (epidemiological processes) happens (Fig. 2).

### Epidemiological processes

The epidemiological processes is a dynamic SEIR compartmental model which considers that susceptible persons (S) are HAV sero-negative or unvaccinated persons. A fraction of susceptibles of age a (age specific vaccination rate) will leave, after vaccination, the susceptible compartment (S) and move to the recovered compartment (R). The proportion of vaccinated individuals who acquired a lifelong immunity is assumed to be equal to e (i.e. the vaccine efficacy). The remaining susceptibles of age i may become infected through contact with an infectious individual of age j (i < =n and j < =n, where n is the maximum age), that new case is proportional to all possible contact between infectious and susceptible individuals (i.e. susceptibles and infectious individuals are supposed to be homogeneously distributed and that a susceptible individual became exposed after a physical contact with an infectious individual, Law of mass action).

Each compartment is categorized into k age groups defined by the age intervals *a*_*k* – 1_, *a*_*k*_, where *k* = 1, 2, …, *n* is the age in years and n is the maximum age.

For *k* = 1, …, *n*, let *x*_*k*_, *y*_*k*_, *i*_*k*_, *z*_*k*_, *u*_*k*_, *r*_*k*_ and *w*_*k*_ are the fraction of susceptible, exposed (not yet infectious), infectious (asymptomatic and symptomatic), recovered and dead persons at age k and time t (i.e. *x*_*k*_ + *y*_*k*_ + *z*_*k*_ + *u*_*k*_ + *r*_*k*_ + *w*_*k*_ = 1 and *i*_*k*_ = *z*_*k*_ + *u*_*k*_).

As hepatitis A is transmitted by both symptomatic and asymptomatic sub-populations, the incidence of the disease in k-th-group is given by:

$$ {\sum}_{j=1}^n{\beta}_{kj}{x}_k{i}_j, $$

Where (*β*_*kj*_)_*n* × *n*_ is the contact matrix with *β*_*kj*_ ≥ 0, ∀ *k*, *j* ∈ {1, …, *n*}, which defines the disease transmission coefficient between susceptible and infectious. The estimation of the contact rate was based on the method explained by Hethcote [29, 30].

Then, the epidemiological model can be state to the following system of differential equation:

For a population without vaccine, we have

2 $$ {\displaystyle \begin{array}{c}\frac{d{x}_k(t)}{dt}=-\sum \limits_{j=1}^n{\beta}_{kj}{x}_k(t)\left({z}_j(t)+{u}_j(t)\right)\\ {}\frac{d{y}_k(t)}{dt}=\sum \limits_{j=1}^n{\beta}_{kj}{x}_k(t)\left({z}_j(t)+{u}_j(t)\right)-\delta {y}_k(t)\\ {}\frac{d{z}_k(t)}{dt}=\left(1-\alpha \right)\delta {y}_k(t)-{\sigma}_k{z}_k(t)\\ {}\frac{d{u}_k(t)}{dt}=\alpha \delta {y}_k(t)-{\sigma}_k{u}_k(t)\\ {}\frac{d{r}_k(t)}{dt}={\sigma}_k{z}_k(t)+\left(1-{\varepsilon}_k\right){\sigma}_k{u}_k(t)\\ {}\frac{d{w}_k(t)}{dt}={\varepsilon}_k{\sigma}_k{u}_k(t)\end{array}} $$

Where 1/*δ* is the latency period, 1/*σ*_*k*_ is the average duration of infectivity by age, *ε*_*k*_ is the death rate due to HAV and *α* is the probability that an infection is icteric.

When vaccination is introduced at age *a*, it is introduced once at the beginning of the years, and we have,

$$ \Big\{{\displaystyle \begin{array}{cc}{x}_a& =\left(1-\mu \right){x}_a\\ {}{r}_a& ={r}_a(t)+\mu {x}_a\end{array}}\operatorname{} $$

Where, *μ* is the vaccine efficacy.

### Demographic processes

Generally, the solving of a set of ordinary differential equations (ODE) is not easy and depends on specific assumption made for the different model parameters. In this work, to reduce an ODE model to a more solvable and workable model, we consider *k* compartmental model representing *k* age groups and using continuous transition from one age group to the next. We use a realistic age structured model which allows individuals to change status from different class (S,E,I,R,D) during 1 year after which they instantaneously move to the next age group.

In Step2, we took an integration time of period equal to 1. However the results of the system are sensitive to the integration time. On the other hand, the time of integration depends on the way in which sickness spreads in the population.

The model consists of the following two-step iteration: Assuming 1 yearage groups. Let {*x*_*i*_(*t*), *y*_*i*_(*t*), *z*_*i*_(*t*), *u*_*i*_(*t*), *r*_*i*_(*t*), *w*_*i*_(*t*)} denote the fraction of susceptible, exposed (not yet infectious), infectious (asymptomatic and symptomatic), recovered and dead persons of age *i* = 1, …, *n* at time *t* (in years).

### Step1

Given initial values

$$ \left\{{x}_i(t),{y}_i(t),{z}_i(t),{u}_i(t),{r}_i(t),{w}_i(t)\right\}=\left\{{x}_i\left({t}_0\right),{y}_i\left({t}_0\right),{z}_i\left({t}_0\right),{u}_i\left({t}_0\right),{r}_i\left({t}_0\right),{w}_i\left({t}_0\right)\right\} $$

For *i* = 1, . . , *n*, solve the following set of system EDO (2) to obtain {*x*_*i*_(*t* + 1), *y*_*i*_(*t* + 1), *z*_*i*_(*t* + 1), *u*_*i*_(*t* + 1), *r*_*i*_(*t* + 1), *w*_*i*_(*t* + 1)} after 1 year, *i* = 1, . . , *n*.

### Step2

Individuals are then shifting by 1 year:

$$ {\displaystyle \begin{array}{c}\left\{{x}_{\left(i+1\right)}\left(t+1\right),{y}_{\left(i+1\right)}\left(t+1\right),{z}_{\left(i+1\right)}\left(t+1\right),{u}_{\left(i+1\right)}\left(t+1\right),{r}_{\left(i+1\right)}\left(t+1\right),{w}_{\left(i+1\right)}\left(t+1\right)\right\}\leftarrow \\ {}\left\{{x}_i\left(t+1\right),{y}_i\left(t+1\right),{z}_i\left(t+1\right),{u}_i\left(t+1\right),{r}_i\left(t+1\right),{w}_i\left(t+1\right)\right\}\\ {}i=1,..,n-1\end{array}} $$

And

$$ {\displaystyle \begin{array}{c}\left\{{x}_1\left(t+1\right),{y}_1\left(t+1\right),\kern0.5em {z}_1\left(t+1\right),{u}_1\left(t+1\right),{r}_1\left(t+1\right),{w}_1\left(t+1\right)\right\}=\\ {}\left\{{x}_1(0),{y}_1(0),\kern0.5em {z}_1(0),{u}_1(0),{r}_1(0),{w}_1(0)\right\}\end{array}} $$

When the vaccine is given at age a, *x*_*a*_(0) is replaced by (1 − *μ*)*x*_*a*_ (0) and *r*_*a*_(0) is replaced by *r*_*a*_(0) + *μx*_*a*_(0).

This process is iterated throughout the time period of interest as previously published [31].

### Model parameters

Demographic data and population size were obtained from the Tunisia National Institute of Statistics (TNIS) and from the 2004 and 2014 census. Epidemiological data were obtained from the current seroprevalence study and from the literature. Definitions and value of parameters are given in Table 1.

**Table 1:** Parameter values by age class, and corresponding references

Parameter	Meaning	Value	Relevant references
b	Birth rate	799 person per year	Estimated from TNIS
*ξ*	Age specific non_HAV death rate	172 per 10,000 person	Estimated from TNIS
1/*δ*	Mean duration of latent period	14 days	[30, 31]
$\frac{1}{\sigma_i}\kern0.75em i=1..n$	Age-specific mean duration of infectious period	(see Appendix)	Estimated
*λ*_*i*_, *i* = 1. . *n* (Used to estimate *β*_*ij*_)	Age-specific true force of infection	(see Appendix)	Estimated (Appendix)
*ε*_*i*_, *i* = 1. . *n*	Age-specific death rate (attributable to HAV infection in symptomatic cases)	(see Appendix)	Estimated
*μ*	Vaccine efficacy	97%	[32, 33]
*N*_*i*_, *i* = 1. . *n*	Population sizes of age class	(see Appendix)	Estimated from total population of the study region (Thala)

## Results

### Seroprevalence of anti-HAV, deduced incidence and force of infection

**Fig. 3:**
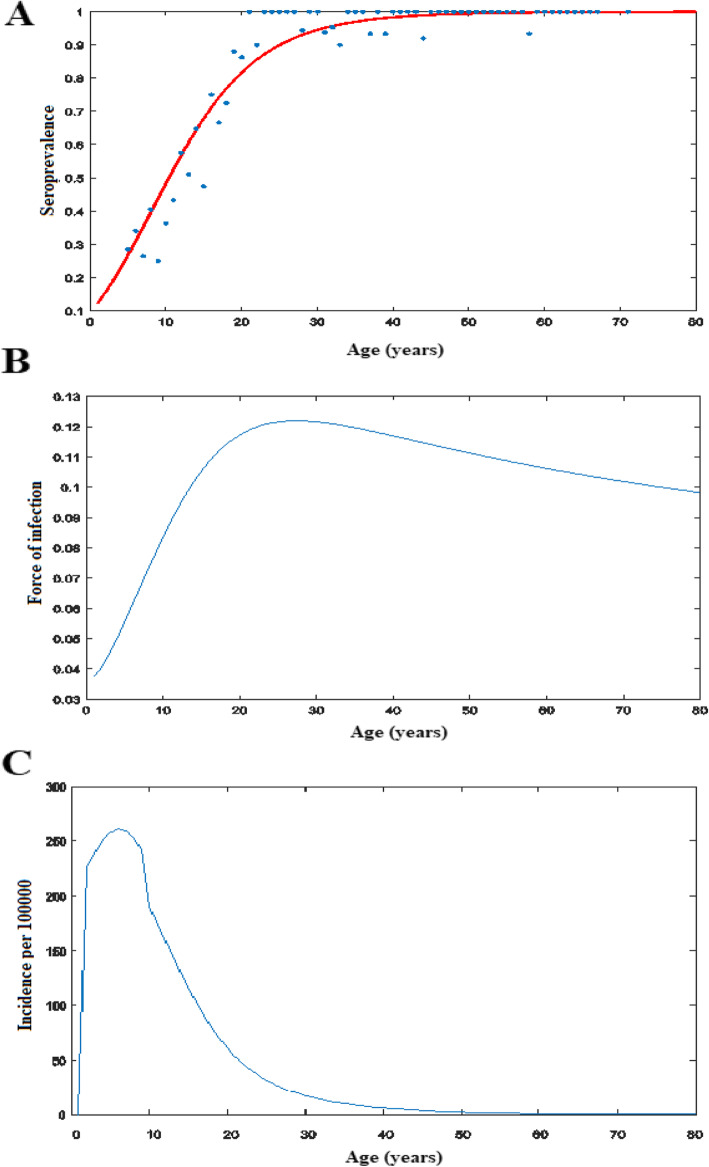
Hepatitis A virus age-specific profiles among Thala population. **a** Seroprevalence estimated from HAV age specific seroprevalences study results according to laboratory and demographic data and adjusted by the model. **b** Force of infection estimated (modeled with logistics link function). **c** Incidence of HAV infections, the model predicted- reported incidence in all age groups (given by I(a) = z(a) + u(a))

Figure 3a shows the IgG anti-HAV seroprevalence according to age groups. A rapid increase is noted during infancy and adolescence: 47% of subjects aged less than 10 years are infected, the prevalence increases to 77% in subjects aged 15 years and reaches 97.1% in subjects aged 30 years. Figure 3b shows the force of infection, estimated according to age. The force of infection is highest in children and young adults aged 10 to 30 years; it remains high with a slight decrease in older ages. Fig. 3c shows that the incidence of infection declines with increasing age. It is relatively high in children aged 1–15 years and then declines rapidly to become very low in adults aged 30 to 80 years.

### Vaccine protection assumptions

Three scenarios were considered. Scenario1 consists in the introduction of two doses of HAV vaccine: a systematic vaccination at 12 months and a catch-up vaccination at 6 years of age during a period of 6 years. Scenario2 consists in the introduction of one dose at 12 months of age and Scenario3 in the introduction of one dose at 6 years of age. Vaccine efficacy was assumed to be 97%, with a lifelong protection.

**Fig. 4:**
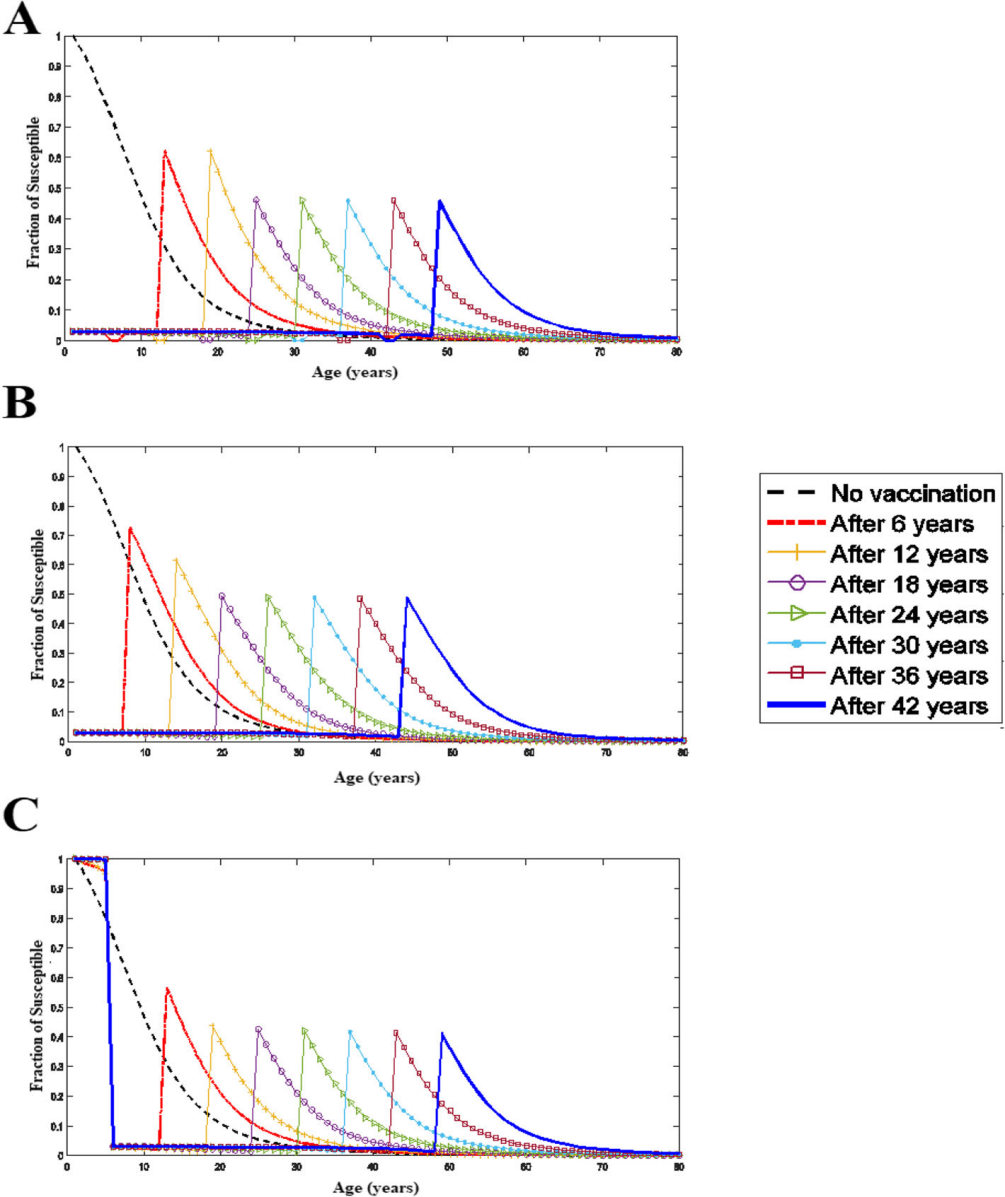
Distribution of susceptible by age at 6, 12, 18, 24, 30 36 and 42 years for scenarios 1, 2 and 3. The black curve defined the fraction of susceptible without vaccination. The other curves defined the fraction of susceptible with vaccination after 6 years up to 42 years. **a** First scenario: Vaccination at the age of 12 months and at the age of six during a period of 6 years. After this period, the HAV vaccine will be given only for those aged 12 month. **b** Second scenario: One dose at the age of 12 months (**c**) Third scenario: One dose at 6 years of age

**Fig. 5:**
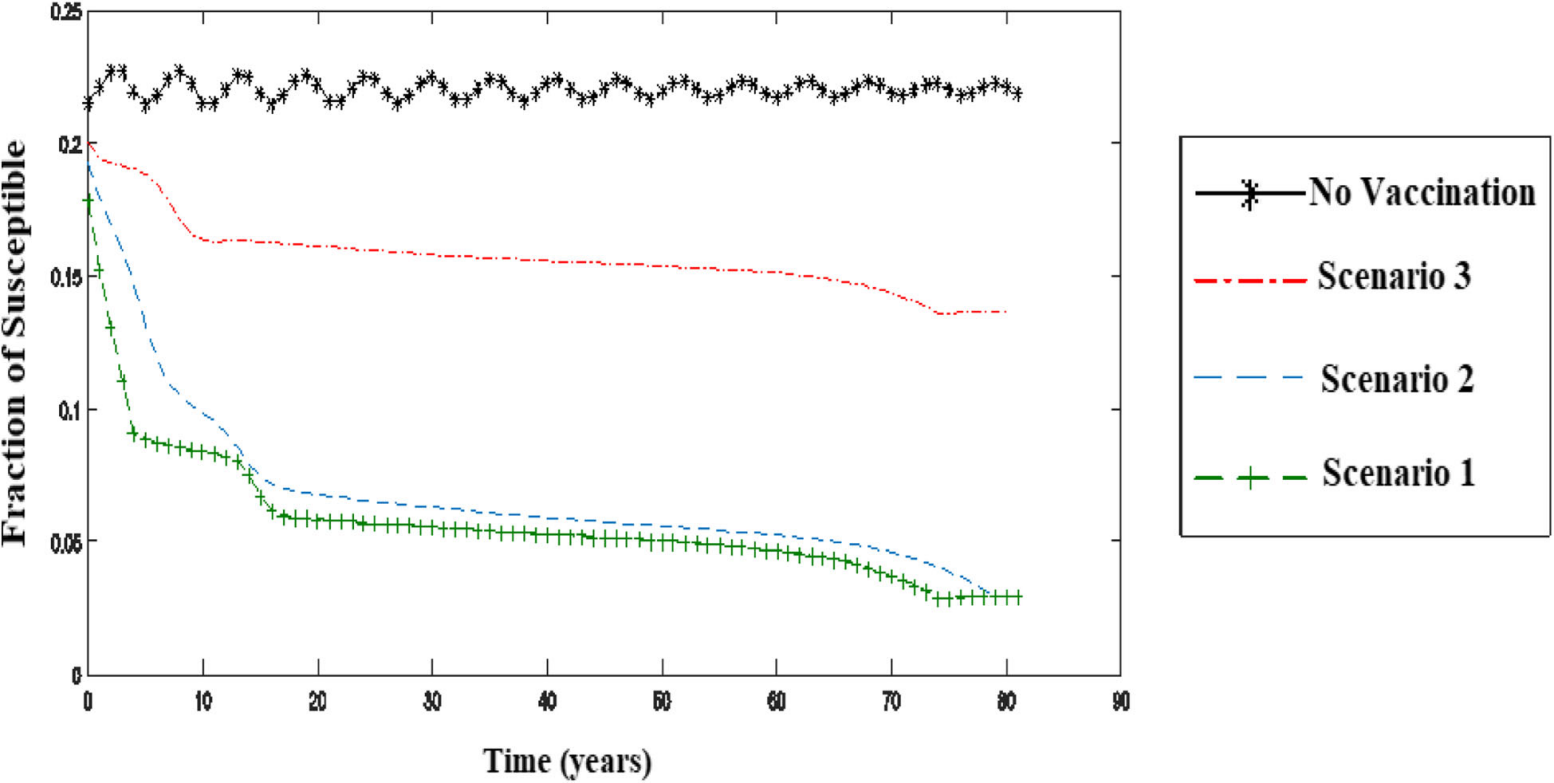
Time evolution of susceptible fraction upon start of vaccine strategies. Scenario 1: Vaccination at the age of 12 months and at the age of six during a period of 6 years. Scenario 2: One dose at the age of 12 months. Scenario 3: One dose at 6 years of age

**Fig. 6:**
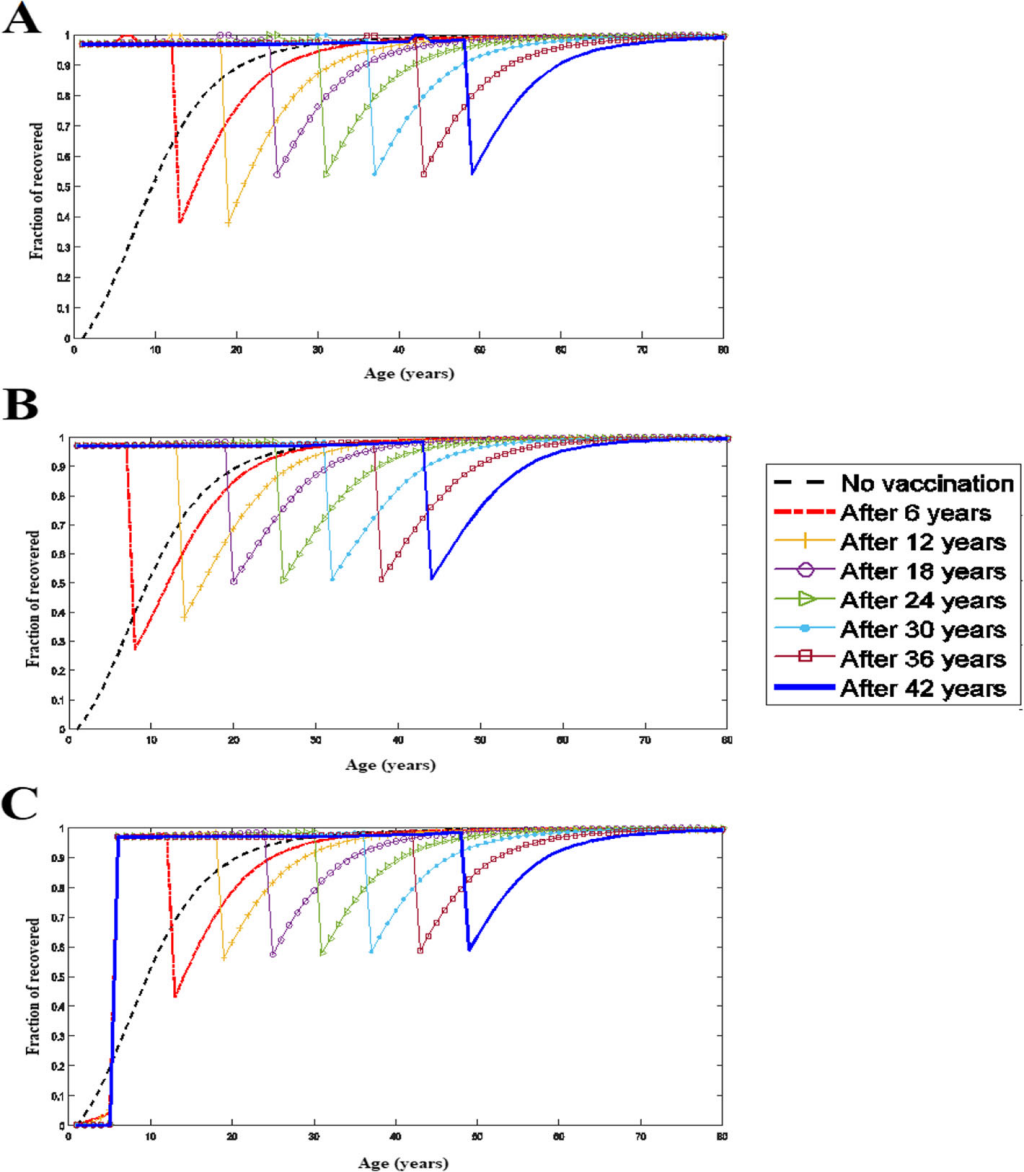
Distribution of recovery by age at 6, 12, 18, 24, 30 36 and 42 years for scenarios 1, 2 and 3. The black curve defined the fraction of recovered without vaccination. The other curves defined the fraction of recovered with vaccination after 6 years up to 42 years. **a** First scenario: Vaccination at the age of 12 months and at the age of six during a period of 6 years. After this period, the HAV vaccine will be given only for those aged 12 month. **b** Second scenario: One dose at the age of 12 months (**c**) Third scenario: One dose at 6 years of age

We simulated the entire population structured by age and epidemiological stage. Figures 4, 5 and 6 show the distribution of the population over time and by proportions of susceptibles or recovery. In Figs. 4 and 6, more precisely, we followed a distribution of the population by age over time and for this distribution we calculated proportions of susceptibles and recovery by age, respectively. For example, the “after 12 years” curve in Fig. 4, corresponds to the distribution of the population by age, 6 years after the “after-6-years” curve. We have done the simulations for the different scenarios. The choice to represent only the cohort at ages 6, 12, 24, 30, ... is purely arbitrary. Figures in 3D were made (data not shown), however a 2D representations with an age distribution of the populations were more explanatory.

Figures 4 and 5 show vaccine efficacy, as assessed by the time evolution of the fraction of susceptible, according to the 3 scenarios described above and in comparison with the base case (absence of vaccination). The Curves “after-n-years”, in Fig. 4a, b and c, show for each scenario, the fraction of susceptible remained by age distribution after each 6-years-period following vaccination. For example for scenario 1 (i.e. when both 1 year-aged and 6 years-aged children are vaccinated), the red curve in Fig. 4a shows that after the first 6 years, individuals aged less than 12 years will be immunized and those aged 13 to 23 years will have the highest proportion of HAV infection susceptible than older persons. The yellow curve represents the repartition of the fraction of susceptible after 12 years of vaccination, shows that individuals aged 19 to 29 years will have the highest proportion of HAV infection susceptible while persons aged 1 to 18 years will be immunized. The purple curve represents the repartition of the fraction of susceptible after 18 years of vaccination, shows that individuals aged 25 to 33 years will have the highest proportion of HAV infection susceptible while persons aged 1 to 24 years will be immunized, etc. Similarly to Figs. 4 and 6 shows the evolution with time of the fraction of recovered according to the 3 scenarios and without vaccination. Therefore, in each scenario (Fig. 6a, b or c) the curves represent the fraction of recovered after each 6-years period following vaccination; each curve shows the fraction of immunized persons by vaccination and naturel infection. As in Fig. 4a, for scenario 1 (i.e. when both 1 year-aged and 6 years-aged children are vaccinated), the red curve in Fig. 6a shows that after the first 6 years, individuals aged 1 to 12 years (immunized by vaccination) and more than 25 years (immunized after naturel infection) acquire almost all a lifelong immunization (they are not susceptible to the infection, fraction of recovered ≥0.9). Individuals aged 13 to 23 years are the individuals susceptible to HAV infection. A fraction of these individuals will contact the virus and develop immunity, with a rate of recovered ranging from 0.4 to 0.8 (Fig. 6a). The yellow curve in Fig. 6a shows as in Fig. 4a that individuals aged 1 to 18 years (immunized by vaccination) and more than 31 years (immunized after naturel infection) are almost all not susceptible to the infection (fraction of recovered ≥0.9) and that susceptible individuals are those aged 19 to 29 years, etc.

According to our model, the 3 scenarios lead to a significant reduction of the fraction of susceptibles which becomes negligible with time for Scenario1 and Scenario2 (Figs. 4 and 5). However, best results are obtained with Scenario1 where the fraction of susceptible becomes negligible after the first 6 years while it decreases but remains important up to 12 years according to Scenario2 and Scenario3. As compared to Scenario2, vaccination at school entry (Scenario3, Fig. 4c) induces a more rapid decrease of the fraction of susceptible among school attendants and adolescents after the first 6 years of vaccination but keeps a fraction of susceptible among children less than 6 years of age. This also appears in Fig. 6, while applying vaccine strategies 1 and 2 will increase significantly the number of recovered by time, with better results for Scenario1, the fraction of recovered will decrease but will remain important and almost the same after 12 years of vaccination in Scenario3. In the case without vaccination, the observed oscillations in Fig. 5 are due to the density dependence term in the Eqs. 2. S(t) oscillates around the equilibrium value (symmetric oscillations) and as time passes the magnitude of the oscillations decreases up to the point at which s(t) reaches the endemic equilibrium fraction.

## Discussion

This study describes epidemiological patterns of HAV infection and predicts the impact of vaccination strategies in a region from central-west Tunisia that accounts among the regions with lowest economic level in the country. Anti-HAV prevalence by age-groups showed a rapid increase during childhood and adolescence. Starting from 31.5% in children aged 5–9 years, it increases with age to reach 50.0, 68.1 and 96.9% in peoples aged 10–14, 15–19 and 20–29 years, respectively. In fact, previous studies conducted in Tunisia and in Jordon and Nicaragua, countries having high/ intermediate HAV endemicity level, showed also that the seroprevalence rate of HAV is high among adolescence and it can reach up to 100% especially in advanced ages [20, 21, 34, 35]. These results classify the study region as of intermediate endemicity, according to the World Health Organization (WHO) criteria: < 90% at 10 years and ≥ 50% at 15 years of age [36]. These results also suggest the transition pattern of HAV from high to intermediate endemicity, probably for the whole of Tunisia, previously classified among countries with high endemicity [19]. Similar epidemiological changes, associated with improved living conditions, are now being observed in many developing countries from the MENA region, such as Algeria, Kuwait, Saudi Arabia, the Emirates and Egypt [13, 14, 37–40] as well as others countries in Africa, Asia, Europe and Latin America [10–16, 34, 35, 41–48]. Since older patients are usually symptomatic, the higher occurrence of infection during adolescence and adulthood results in an increased number of symptomatic cases and may lead to serious outbreaks. Therefore, vaccination stands out as the best measure to prevent hepatitis A and is now recommended by WHO for countries showing transition from high to intermediate endemicity. The high effectiveness of hepatitis A vaccines was shown in reducing disease burden and HAV outbreaks [15, 41, 42]. Many countries such as Argentina, Bahrain, Brazil, China, Greece, Panama, the US and Uruguay; as well as regions of Belarus (Minsk City), Canada (Quebec), Italy (Puglia) and Spain (Catalonia) had introduced HAV vaccine in their universal immunization programs [40, 43, 49–56]. As of May 2019, 34 countries used or were planning to introduce hepatitis A vaccine in routine immunization of children nationally [57]. In Tunisia, during the past 3 years, many cases of hepatitis A were notified to the ministry of health; most of them were children attending schools and aged over 6 years. Field investigations showed that the majority of the schools where these cases occurred have bad sanitary conditions in terms of access to safe drinking water and the availability of water in the sanitary blocs. In addition to measures aiming the improvement of hygiene in schools, systematic vaccination strategies against HAV were also considered.

At the international level, both inactivated and live attenuated vaccines are now available. Inactivated vaccines were developed since 1992. A complete vaccination schedule consists of 2 doses administered at 6–36 months interval [36, 58], although several studies showed that a single dose is sufficient [32, 59, 60]. Inactivated vaccines generally produce comparable immune responses with a protective efficacy of 94% [33, 36]. They have been introduced in the national immunization program of many countries: two doses in Mongolia (14 months and 2 years), Turkey (18 and 24 months), Israel (18 and 24 months), Uruguay (15 and 21 months), Bahrain (15 months and 2 years), Saudi Arabia (15 months and 2 years), Panama (12 and 18 months), Kazakhstan (2 and 2.5 years) and Qatar (12 and 18 months) and a single dose in Brazil (15 months), Argentina, Colombia and Mexico, for children aged 12 months in the three latter countrie s[61]. Live attenuated HAV vaccines were more recently developed and are licensed for a single subcutaneous administration in children aged ≥1 year. They provide a protective efficacy of 95% and are mainly used in China and few other countries (India, Thailand, Philippines, Guatemala, Bangladesh) [62].

Presently, the WHO encourages introduction of HAV vaccine in the immunization schedule of countries with intermediate endemicity and in countries experiencing increased morbidity and mortality [36]. In the United States of America, the Advisory Committee on Immunization Practices recommends vaccination of children aged 12–23 months together with catch-up vaccination of older children and vaccination of persons at high risk [20]. In Tunisia, inactivated vaccines were available with a relatively expensive cost and used for around 10% of children vaccinated in the private sector. In the past few years, inactivated vaccine was given to the contacts of confirmed cases to limit the spread of the virus during outbreaks and, since October 2018, it is given to 6 years-old children at school entry. However, other vaccination strategies are still under discussion within the NITAG and the results of the present study will help to choose the best strategies. Although a strategy including a first dose at 12 months of age and a one-time catch-up vaccination for children aged 1–6 years would be the most effective, this was considered as non-feasible for financial considerations. The only possible schemes would consist in the introduction of only one dose or, at most, of two doses. According to the current national vaccination schedule, the best time to deliver this single dose of vaccine may be 12 months of age, together with the 1st dose of Measles/Rubella vaccine, or 6 years at school entry together with the Polio booster vaccine. Due to the recent outbreaks in school-aged children, it was proposed to deliver this vaccine at school entry to rapidly reduce the infection rate among school attendees and interrupt the epidemic trend of the disease. In this study, we developed an age structured epidemiological model to assess the impact of the introduction of vaccination with 3 scenarios: a single dose at 12 months, a single dose at 6 years of age, and one dose at 12 months with a dose at 6 years of age during 6 years. Vaccinating at 12 months of age (scenario 2) is the standard scheme and the most widely used scenario in the world. Vaccination at 6 years of age (scenario 3) is more efficient to interrupt rapidly the epidemic trend among school attendants. Scenario 1 combines the two scenarios allowing a systematic protection of all neonates starting from the earliest recommended age for HAV vaccine and a vaccination at school entry to rapidly reduce the burden of the disease in schools. Scenario 1 will thus offer a catch-up vaccination of children aged 1 to 6 years during a period of 6 years. Our results showed that the three strategies can significantly reduce the incidence of hepatitis A infection and that the reduction will be faster with Scenario1. With regard to the vaccinations strategies using only one dose, our results show that vaccination at school entry induces a more rapid decrease of the fraction of susceptible among school attendants and adolescents but keeps a fraction of susceptible among children less than 6 years of age which will not provide an optimal long-term efficacy of the vaccination strategy.

## Conclusion

The present study confirms the transition to intermediate endemicity levels in a region of Central-West Tunisia with lowest socioeconomic conditions in the country, and suggests the same transition in the other parts of the countries that have similar conditions and also in regions with better socioeconomic and sanitary conditions where HAV prevalences should be even lower. These results urge national authorities to undertake appropriate prevention measures to avoid enhanced hepatitis A morbidity and mortality. The models presented herein offer mid and long-term projection on the efficacy of HAV vaccination and might facilitate national decisions with regards to possible vaccinations strategies. However, this study has to be followed up by economic evaluation before submission of the evidence to NITAG.

## Supplementary information

**Additional file 1.**

## Data Availability

The data that support the findings of this study are available from the corresponding author upon reasonable request.

## Abbreviations

HAV: Hepatitis A virus
NITAG: National Immunization Technical Advisory Group
Anti-HAV: HAV antibodies
SEIR: Susceptible-Exposed-Infectious-Recovered
ODE: Ordinary differential equations
TNIS: Tunisia National Institute of Statistics
WHO: World Health Organization
