# Supplementary material for: Hepatitis a virus infection in Central-West Tunisia: an age structured model of transmission and vaccination impact
Source: BMC Infect Dis. 2020 Aug 26;20:627. doi: 10.1186/s12879-020-05318-7 (PMC7477833; doi:10.1186/s12879-020-05318-7)
Supplement: Supplementary file 1 — Additional file 1. [file 12879_2020_5318_MOESM1_ESM.docx]

**Appendix: Estimation of FOI: λ(a)**

We assumed that the disease is in a stable state population, which means that the age distribution of the population is independent of time. So, the probability $\pi\left( a \right)=1-exp\left( -\int_{0}^{a} \lambda\left( a \right)da \right)$ is the seroprevalence of the disease (probability of being infected before age a). We have,

|  | $\lambda\left( a \right)=\frac{\pi'\left( a \right)}{1-\pi\left( a \right)}$ | (2) |
| --- | --- | --- |

The seroprevalence, $\pi\left( a \right)$ is parameterized using the data of the seroprevalence of hepatitis A virus antibodies in a rural setting in central-west Tunisia (Thala). In general, $\pi\left( a \right)$ is modeled as $\pi\left( a \right)=g^{-1}\left( \eta\left( a \right) \right)$ where $\eta$ is a linear predictor and $g$ is a link function. We use the link function so-called logistics defined by

|  | $g\left( t \right)=log\left( \frac{t}{1-t} \right)0<t<1$ | (3) |
| --- | --- | --- |

but we can use other link function like $g\left( t \right)=log\left( -log\left( 1-t \right) \right)$ and $g\left( t \right)=-log\left( 1-t \right)$ (as previously published **[1]**, **[2]**, **[3]**, **[4]**, **[5]** and **[6]**).

Using (3) and the definition of the force of infection (2), we get

$\pi\left( a \right)=\frac{e^{\eta\left( a \right)}}{1+e^{\eta\left( a \right)}}$ and $\lambda\left( a \right)=\eta^{'}\left( a \right)\frac{e^{\eta\left( a \right)}}{1+e^{\eta\left( a \right)}}$

For the choice of predictor for the adjusted model, Royston and Altman **[7]** have presented a fractional polynomial family in the context of binary response and have argued that in practice the Fractional polynomials of order greater than 2 are rarely used. In our work, we suppose that:

|  | $\eta\left( t \right)=\beta_{0}+\beta_{1}t^{p_{1}}+\beta_{2}t^{p_{2}}$ | (4) |
| --- | --- | --- |

Model parameters $\beta_{0},\beta_{1},\beta_{2},p_{1}$ and $p_{2}$ were estimated by the likelihood method as previously published **[8]**.

Once we had this estimate (the estimated parameter), we had to evaluate it quality. For the evaluation, the widespread way was used is the calculation of the deviation which is a measure based on the likelihood function. This method compares the model estimated with a saturate model. The saturate model is a model possessing as much parameter as observation and thus estimating exactly the data: $D=-2\left( L-L_{sat} \right)$, where $L$ and $L_{sat}$ are the likelihood functions maximized for the estimated model and the saturated model. Since the saturated model imposes no restriction on the$\left( a_{i} \right)$, the maximum likelihood in the saturated model is:

|  | $L_{sat}=\sum_{i=1}^{n} m_{i}log\left( \pi\left( a_{i} \right) \right)+\left( 1-m_{i} \right)log\left( 1-\pi\left( a_{i} \right) \right)$ | (5) |
| --- | --- | --- |

The maximum likelihood of our model is

|  | $L=\sum_{i=1}^{n} m_{i}log\left( \hat{\pi}\left( a_{i} \right) \right)+\left( 1-m_{i} \right)log\left( 1-\hat{\pi}\left( a_{i} \right) \right)$  Where  $m_{i}=\left\{ \begin{matrix} 1 & if the individual had an infection before age a_{i} \\ 0 & \mathrm{otherwise} \end{matrix} \right.$ | (6) |
| --- | --- | --- |

And

$$\hat{\pi}\left( a \right)=\frac{e^{\eta\left( a_{i} \right)}}{1+e^{\eta\left( a_{i} \right)}}$$

If the model is adequate, L and $L_{sat}$(5) and (6) will have close values and the deviance will be weak. In general, the deviance follows the chi-square law with n-p degree of freedom where p is the number of parameters estimated in our model.

For the Thala region data set, the second-order fractional polynomial which has a deviance of 79.04 on 59 degrees of freedom. In addition, we have$\beta_{0}=-2.3493$,$\beta_{1}=-0.2$,$\beta_{2}=0.5822$, $p_{1}=0.5$and$p_{2}=0.7$.

For $=1..n$ , The infection period ${1/\sigma}_{i}$, the age-specific death rate attributable to HAV infection in symptomatic case $\varepsilon_{i}$ and the population size of age class $N_{i}$ is defined as follows:

${1/\sigma}_{i}=\left\{ \begin{aligned} 3 week if i\leq9 \\ 2.5 week if i>9 \end{aligned} \right.$

$$\varepsilon_{i}=\left\{ \begin{aligned} 0.001\% \mathrm{if} i\leq14 \\ 0.014\% \mathrm{if} 15\leq i\leq39 \\ 0.109\% \mathrm{if}40\leq i\leq59 \\ 0.736\% \mathrm{if} i\geq60 \end{aligned} \right.$$

$$N_{i}=799e^{-0.0172i}$$

**Reference**

[1] Shkedy Z, Aerts M, Molenberghs G, Beutels P, Van Damme P. Modelling age-dependent force of infection from prevalence data using fractional polynomials. Stat Med. 2006;25(9):1577-91.

[2] Muench H. Catalytic models in epidemiology. New York: Harvard University Press; 1959.

[3] Griffiths DA. A catalytic model of infection for measles. Appl Stat. 1974; 23(3):330-339.

[4] Hens N, Faes C, Aerts M, Shkedy Z, Mintiens K, Laevens H, et al. Handling missingness when modeling the force of infection from clustered seroprevalence data. J Agric Biol Environ Stat. 2007;12(4):498.

[5] Grenfell BT, Anderson RM. The estimation of age-related rates of infection from case notifications and serological data. J Hyg (Lond). 1985;95(2):419-36.

[6] Hens N, Shkedy Z, Aerts M, Faes C, Van Damme P, Beutels P. Modeling infectious disease parameters based on serological and social contact data: a modern statistical perspective (Vol. 63). Springer Science & Business Media; 2012.

[7] Royston P, Altman DG. Regression using fractional polynomials of continuous covariates: parsimonious parametric modelling. Appl Stat. 1994: 429-467.

[8] McCullagh P, Nelder JA.  Generalized linear models (Vol. 37). CRC press; 1989.
